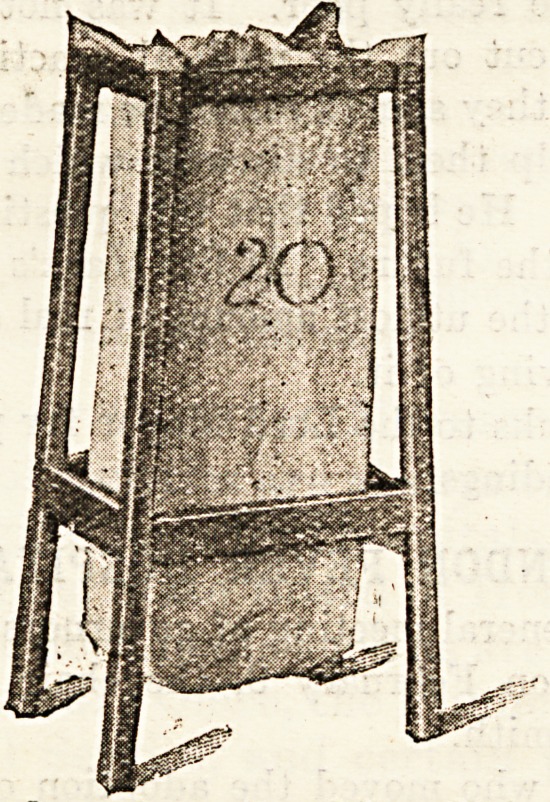# Practical Departments

**Published:** 1906-02-17

**Authors:** 


### PRACTICAL DEPARTMENTS.

### THE FURNISHING OF KING EDWARD VII. SANATORIUM.

The Advisory Committee of the King's Sanatorium invited several firms to tender for the furnishing of wards, nurses' and servants' departments, and to submit complete specimen suites. The final selection has been in favour of Messrs. Heal and Son, Tottenham Court Road, W., and their designs may therefore be regarded as fulfilling the exacting requirements of hygiene, good material and workmanship, commensurate with economy of outlay. The makers take pride in the fact that the furniture is all handmade, which is undoubtedly the safest guarantee of durability and good manufacture.

The ward furniture is made of birch; the surfaces are smooth and polished, and are so designed that dust can find no crevice or corner for lodgment. With this view the wardrobe has a domed roof, while all interior angles of drawers and cupboards are stopped and rounded off. Each article of furniture is well raised from the floor to facilitate cleaning. The washstand top, with shelf beneath and screen at back, and the top of the bedside locker, are all made of glass and are removable: the advantage in preserving cleanliness and in preventing corrosion being obvious. The cupboard of the ward locker has a battened floor and back to ensure ventilation. An interesting feature is the soiled linen stand and bag, which takes the place of the usual linen basket. This bag (which is made of coarse Irish linen) can be removed from the ward to the laundry without the necessity and accompanying risk of disturbing its contents.

The bedstead which has been chosen is the Taunton anti-sagging "Diagna." All open joints and unnecessary excrescences are avoided in the frame of this bedstead, while the springs of the mattress are diagonally arranged instead of straight, thus securing an even distribution of strain, and consequently increased durability. Buffers are fitted at the head of the bedstead to protect it from the wall, and these are made as handles for use in wheeling the bed (which is on rubber-tyred wheels) from the ward to the balcony.

Messrs. Heal & Son are holding an exhibition of this furniture, together with other hospital specialities, including aseptic tables, trolleys, ward cabinets, and bedsteads for many purposes. Seeing that their manufactures have been approved by the highest medical authority, those concerned in the efficient equipment of hospitals, sanatoria, etc., will no doubt pay a visit to this exhibition, the entrance to which is at the manufacturers' premises in Tottenham Court Road, W.

## Figures and Tables

**Figure f1:**